# Serum cytokine profiles predict survival benefits in patients with advanced hepatocellular carcinoma treated with sorafenib: a retrospective cohort study

**DOI:** 10.1186/s12885-017-3889-x

**Published:** 2017-12-19

**Authors:** Tomoyuki Hayashi, Taro Yamashita, Takeshi Terashima, Tsuyoshi Suda, Hikari Okada, Yoshiro Asahina, Takehiro Hayashi, Yasumasa Hara, Kouki Nio, Hajime Sunagozaka, Hajime Takatori, Kuniaki Arai, Yoshio Sakai, Tatsuya Yamashita, Eishiro Mizukoshi, Masao Honda, Shuichi Kaneko

**Affiliations:** 1 Department of Gastroenterology, Kanazawa University Graduate School of Medical Science, 13-1 Takara-Machi, Kanazawa, Ishikawa ZIP 920-8641 Japan; 2 Department of General Medicine/Department of System Biology, Kanazawa University Hospital/Kanazawa University Graduate School of Medical Science, 13-1 Takara-Machi, Kanazawa, Ishikawa ZIP 920-8641 Japan

**Keywords:** Hepatocellular carcinoma, Sorafenib, Hepatic arterial infusion chemotherapy, Cytokine, Chemokine, Growth factor

## Abstract

**Background:**

Sorafenib is a multiple receptor tyrosine kinase inhibitor known to prolong overall survival in patients with advanced hepatocellular carcinoma (HCC). Predicting this drug’s survival benefits is challenging because clinical responses are rarely measurable during treatment. In this study, we hypothesized that serum cytokines levels could predict the survival of advanced HCC patients, as sorafenib targets signaling pathways activated in the tumor stromal microenvironment and potentially affects serum cytokine profiles.

**Methods:**

Of 143 patients with advanced-stage HCC, 104 who were recruited between 2003 and 2007 received hepatic arterial infusion chemotherapy (HAIC) that mainly targets tumor epithelial cells at S-phase (cohort 1); additionally, 39 recruited between 2010 and 2012 received sorafenib, which primarily targets the stromal vascular endothelial cells. Serum samples were collected and aliquoted prior to the treatment. Serum EGF, bFGF, HGF, IFN-γ, IL-10, IL-12, IL-2, IL-4, IL-5, IL-6, IL-8, IP-10, MIG, PDGF-BB, SCF, SDF1, TGF-β, TGF-α, TNF-α, and VEGF-A were measured via enzyme-linked immunosorbent assays. The Modified Response Evaluation Criteria in Solid Tumors were used to assess tumor responses.

**Results:**

The median survival time of HCC patients in cohorts 1 (HAIC-treated) and 2 (sorafenib-treated) were 12.0 and 12.4 months, respectively. Kaplan-Meier analysis revealed no significant survival differences between the 2 groups. Patients who survived more than 2 years after sorafenib treatment exhibited higher serum levels of IL-10, IL-12, TNF-a, IL-8, SDF-1, EGF, PDGF-BB, SCF, and TGF-α. Furthermore, cohort 2 patients with higher serum IL-5 (>12 pg/mL), IL-8 (>10 pg/mL), PDGF-BB (>300 pg/mL), and VEGF-A (>50 pg/mL) levels achieved longer survival; cohort 1 patients did not. Hierarchical cluster analysis of 6 cytokines robustly enriched for comparison analysis between cohorts 1 and 2 (IL-5, IL-8, TGF-α, PDGF-BB, CXCL9, and VEGF-A) revealed that elevation of these cytokines correlated with better survival when treated with sorafenib but not with HAIC.

**Conclusions:**

Patients who exhibited survival benefits owing to sorafenib treatment tended to present higher serum cytokines levels, potentially reflecting the activation of stromal signaling in the tumor microenvironment. Our study thus introduces novel biomarkers that may identify advanced HCC patients who may experience survival benefits with sorafenib treatment.

**Electronic supplementary material:**

The online version of this article (10.1186/s12885-017-3889-x) contains supplementary material, which is available to authorized users.

## Background

Hepatocellular carcinoma (HCC) is the fifth most common cancer and the second leading cause of cancer death in men worldwide [1]. Sorafenib tosylate is a multikinase inhibitor that has been reported to prolong overall survival (by a median of approximately 3 months) in patients with advanced HCC; however, its response rate is generally <5% according to the Response Evaluation Criteria in Solid Tumors [2, 3]. In contrast, hepatic arterial infusion chemotherapy (HAIC) using 5-fluorouracil and cisplatin is widely used for the treatment of advanced HCC in Japan, to which the patient response rates are generally 20–30%. Nevertheless, HAIC does not significantly improve overall survival compared to sorafenib. The responses of the patients who receive HAIC for treating HCC reportedly predict their overall survival, although it is challenging to make similar predictions prior to the treatment. Thus, novel biomarkers that can predict the outcomes of patients with advanced HCC with respect to current treatment modalities are required [4]. However, no studies thus far have successfully identified useful biomarkers that can predict the clinical responses or overall survival rates in patients who received sorafenib or HAIC for advanced HCC.

Recent evidence suggests that the tumor microenvironment plays a pivotal role in the response to anti-cancer agents and molecular targeted therapies [5–7]. Because sorafenib targets the vascular endothelial growth factor (VEGF) receptor 2 (VEGFR2) expressed in endothelial cells, activation of VEGF signaling may correlate with tumors’ responses to sorafenib treatment. Indeed, a recent study indicated that HCC with *VEGFA* amplification showed better responses to sorafenib [8]. Furthermore, a study evaluating genome alteration in advanced HCC patients who responded to sorafenib found that *FGF3/4*, which may result in the activation of fibroblast growth factor (FGF) signaling in the HCC microenvironment, was amplified [9]. The role of serum cytokines as biomarkers for the prediction of sorafenib responses is intriguing [10]; however, it remains unclear if such cytokines are sensitive enough to reflect the status of the tumor microenvironment, which in turn might affect the clinical response in advanced HCCs. In this study, we investigated 20 serum cytokines and growth factors in patients with advanced-stage HCC; their expression profiles were compared between 2 HCC cohorts of patients who received HAIC and sorafenib, respectively. This was particularly important since the former mainly targets S-phase tumor epithelial cells while the latter targets the stromal vascular endothelial cells.

## Methods

### Patients

There were 143 patients with advanced HCC enrolled in our institute (Kanazawa University); 65 with Barcelona Clinic Liver Cancer stage B, 72 with stage C, and 6 with stage D. All patients had Eastern Cooperative Oncology Group performance status scores of 0/1/2 (Table 1). Among these patients, 104 who were recruited between 2003 and 2007 received interferon and HAIC using 5-fluorouracil (300 mg/m^2^, days 1–5 and 8–12) alone or in combination with cisplatin (20 mg/m^2^, days 1 and 8), according to the inclusion and assignment criteria of our previous study [11]. Because sorafenib was approved for the treatment of advanced HCC in 2009, 39 patients recruited in the years 2010–2012 underwent sorafenib treatment (400 mg twice daily). The ‘Response Evaluation Criteria in Solid Tumors’ rules were used to assess tumor responses according to dynamic computed tomography findings. Sera from all patients were aliquoted and stored at −20 °C prior to treatment. This study was approved by the Institutional Review Board of the Kanazawa University [The ethical approval reference number: 2015–257 (2142)], and all patients provided written informed consent for inclusion in the study.

**Table 1:** Baseline disease characteristics

	Cohort 1: HAIC(*n* = 104)	Cohort 2: Sorafenib(*n* = 39)	*P*-value^a^
Age (y), median, range	65.0 (40–82)	67.0 (37–83)	0.306
Gender(Male: Female)	86:18	32:7	0.928
Child-Pugh score	6.5	5.9	0.028
Child-Pugh A	60	31	0.042
Child-Pugh B	40	8	
Child-Pugh C	4	0	
Performance status (0/1/2)	91/11/2	37/1/1	0.301
Prior treatment (surgical resection)	5.2% (20)	33.3% (13)	0.075
Prior treatment (radiofrequency ablation)	51.0% (53)	53.8% (21)	0.759
Prior treatment (TACE/TAE)	51.9% (54)	76.9% (30)	0.007
Size of dominant tumour (mm)	32.3 ± 21.2	42.0 ± 27.9	0.066
Multiple tumour	69.2% (72)	71.8% (28)	0.766
AFP (ng/ml) mean ± SE	13,110.5 ± 5278.7	3438.7 ± 1123.3	0.259
AFP-L3 (%) ± SE	28.6 ± 2.7	38.8 ± 5.4	0.073
DCP (mAU/ml) ± SE	21,194.0 ± 6814.7	5418.4 ± 2896.5	0.161
Vascular invasion (%)	35.6% (37)	17.9% (7)	0.042
Distant metastasis (%)	10.6% (11)	35.9% (14)	<0.001
BCLC stage (B/C/D)	47/51/6	18/21/0	0.303
Tumor responses by mRECIST (CR / PR)	34.6% (36)	2.6% (1)	<0.001

*AFP* alpha-fetoprotein, *AFP-L3* lectin-reactive AFP, *BCLC* Barcelona-Clínic Liver Cancer, *CR* complete response, *DCP* des-gamma carboxyprothrombin, *HAIC* hepatic arterial infusion chemotherapy, *PR* partial response, *SD* standard deviation, *SE* standard error, *TACE/TAE* transcatheter arterial chemoembolization/transcatheter arterial embolization)

^a^χ^2^ tests

### Serum profiles

A total of 20 cytokines, including serum epidermal growth factor (EGF), basic FGF (bFGF), hepatocyte growth factor (HGF), interferon (IFN)-γ, interleukin (IL)-10, IL-12, IL-2, IL-4, IL-5, IL-6, IL-8, chemokine (C-X-C motif) ligand (CXCL)-9, CXCL10, platelet-derived growth factor (PDGF)-BB, stem cell factor (SCF), stromal cell-derived factor 1 (SDF1), transforming growth factor (TGF)-α, transforming growth factor (TGF)-β, tumor necrosis factor (TNF)-α, and vascular endothelial growth factor (VEGF-A), were measured in duplicate by a multiplex biometric enzyme-linked immunosorbent assay (ELISA)-based immunoassay (Bio-Plex, Bio-Rad Laboratories, Inc. Hercules, CA) according to the manufacturer’s protocol. Serum alpha-fetoprotein (AFP), lectin-reactive AFP (AFP-L3), and des-gamma carboxyprothrombin (DCP) were measured by conventional methods using commercially available assays at the Kanazawa University Hospital.

### Statistical analysis

Student *t* tests or χ^2^-tests were performed using the GraphPad Prism software version 5.0.4 (GraphPad Software) to compare the 2 groups. Kaplan-Meier survival analyses with log-rank tests were also performed using the GraphPad Prism software. Hierarchical clustering analysis was performed using the Genesis software (v. 1.6.0 beta).

## Results

### Baseline cohort characteristics

A total of 143 advanced-stage HCC patients were included in this study. Patients who received HAIC were recruited between 2003 and 2007 (cohort 1), whereas those who received sorafenib were recruited between 2010 and 2012 (cohort 2); sorafenib was only introduced in Japan in 2009. The profiles of the patients in both cohorts are shown in Table 1. The patients in both cohorts were similar with respect to age and sex, as well as to AFP, AFP-L3, and DCP levels. However, the number of patients with Child-Pugh classes B and C in cohort 1 were significantly higher than those in cohort 2 (*P* = 0.042). This might be related to the inclusion of 4 decompensated Child-Pugh C cirrhosis patients with advanced HCCs, because HAIC was successfully used in such patients [11]. The frequency of vascular invasion was higher in cohort 1 (*P* = 0.04), whereas that of distant metastasis was higher in cohort 2 (*P* < 0.001).

**Fig. 1:**
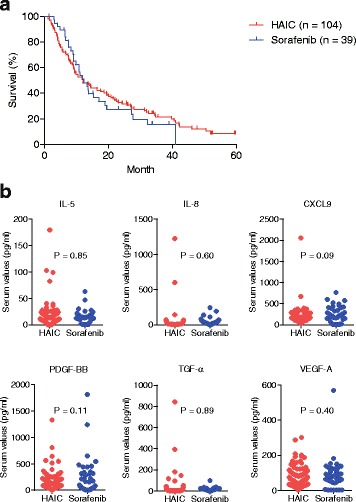
Characteristics of hepatocellular carcinoma (HCC) patients in the 2 cohorts. **a** Kaplan-Meier survival analysis of HCC patients in the 2 cohorts. The median survival time of HCC patients who received hepatic arterial infusion chemotherapy (HAIC) and sorafenib was 12.0 months and 12.4 months, respectively. There were no significant survival differences between the two cohorts (log-rank test, *P* = 0.65). **b** Serum levels of 6 cytokines (IL-5, IL-8, CXCL9, PDGF-BB, TGF-α, and VEGF-A) were distributed similarly in the sera of HCC patients in cohorts 1 and 2

Clinical responses (complete or partial remission) were observed in 36/104 cases (34.6%) in cohort 1 and 1/39 cases (2.6%) in cohort 2; such outcomes are consistent with previously reported results [2, 3, 11]. Although the baseline characteristics of the 2 cohorts showed some differences, the median survival times of patients who received HAIC and sorafenib were 12.0 months and 12.4 months, respectively. Kaplan-Meier survival analysis revealed no significant survival differences between the 2 cohorts (log-rank test, *P* = 0.65) (Fig. 1a).

Next, we evaluated the expression levels of 20 cytokines using patients’ sera obtained prior to treatment in both cohorts. Because we evaluated the serum profiles of patients in cohort 1 in 2009 and cohort 2 in 2013, we had used different versions of the Bio-Plex assay kits for the analyses; therefore, we first compared the distribution of serum proteins measured in cohorts 1 and 2. Unexpectedly, the distribution of 14 cytokines (EGF, bFGF, HGF, IFN-γ, IL-10, IL-12, IL-2, IL-4, IL-6, CXCL10, SCF, SDF1, TGF-β, and TNF-α) differed between cohorts 1 and 2, suggesting that the improvement of the detection threshold of the Bio-Plex analysis system might bias the analysis. We therefore excluded these 14 cytokines from comparison analysis of the serum profiles between cohort 1 and 2. The remaining 6 cytokines (IL-5, IL-8, CXCL9, PDGF-BB, TGF-α, and VEGF-A) showed no difference in terms of distribution in cohorts 1 and 2 (Fig. 1b). We thus analyzed these cytokines to evaluate their survival-related significance in cohorts 1 and 2.

### Treatment response and serum cytokines

We compared the patients’ serum profiles in cohort 1 based on the clinical responses (complete response/partial response and stable disease/progressive disease) (Table 2). Patients with clinical responses had higher serum SDF1 levels (*P* = 0.02) and lower serum HGF (*P* = 0.04) and IL-4 (*P* = 0.04) levels compared to non-responders. We then classified the HCCs as HGF-high and -low, IL-4-high and -low, and SDF1-high and -low using the median values of non-responders as cutoff values: HGF (500 pg/mL), IL-4 (1 pg/mL), and SDF1 (1 pg/mL). Kaplan-Meier based on the median indicated that patients with high serum IL-4 levels showed significantly poorer prognoses (*P* = 0.03) (Additional file 1: Figure S1).

**Table 2:** Baseline serum cytokines in Cohort 1 (hepatic arterial infusion chemotherapy-treated patients)

Cytokines (pg/mL, mean ± SE)	CR/PR group	SD/PD group	*P*-value^a^
EGF	20.8 ± 3.8	21.6 ± 3.0	0.882
FGF	2.84 ± 1.63	8.40 ± 2.53	0.134
HGF	523.9 ± 50.3	739.8 ± 71.6	0.043
IFN-γ	0.109 ± 0.064	1.343 ± 0.699	0.203
IL-10	0.813 ± 0.169	1.239 ± 0.219	0.193
IL-12 (p70)	8.41 ± 1.17	10.77 ± 1.05	0.164
IL-2	0.297 ± 0.092	1.118 ± 0.568	0.299
IL-4	0.586 ± 0.170	1.213 ± 0.197	0.038
IL-5	17.3 ± 5.5	15.6 ± 2.0	0.718
IL-6	10.90 ± 3.94	9.05 ± 1.65	0.614
IL-8/CXCL8	61.3 ± 37.1	18.7 ± 2.7	0.119
CXCL10	358.3 ± 41.1	359.1 ± 30.3	0.988
CXCL9	176.6 ± 14.0	205.8 ± 30.6	0.502
PDGF-BB	152.5 ± 20.9	193.6 ± 24.3	0.265
SCF	14.1 ± 1.3	16.4 ± 1.0	0.189
SDF-1	60.3 ± 26.3	13.1 ± 5.0	0.022
TGF-β	5.17 ± 3.42	1.64 ± 0.71	0.188
TGF-α	34.0 ± 23.8	14.2 ± 6.3	0.307
TNF-α	1.23 ± 0.31	1.87 ± 0.30	0.184
VEGF-A	65.1 ± 9.4	83.6 ± 6.9	0.116

*CR* complete response, *PD* progressive disease, *PR* partial response, *SD* stable disease, *SE* standard error

^a^Unpaired t-tests

Similarly, we evaluated the serum cytokine levels in HCC patients who received sorafenib treatment. Because such treatment rarely showed clinical responses, we classified patients with HCC into long survivors (overall survival ≥2 years) and non-long survivors (overall survival <2 years) (Table 3). Surprisingly, 9 of 20 cytokines (IL-10, IL-12, TNF-α, IL-8, SDF-1, EGF, PDGF-BB, SCF, and TGF-α) were significantly elevated in long survivors compared to non-long survivors (*P* < 0.05) (Table 3).

**Table 3:** Baseline serum cytokines in Cohort 2 (sorafenib-treated patients)

Cytokines (pg/mL, mean ± SE)	Survival time ≥ 2 years(*n* = 7)	Survival time < 2 years(*n* = 32)	*P*-value^a^
EGF	213.7 ± 99.5	58.3 ± 10.6	0.004
FGF	89.6 ± 46.8	103.4 ± 19.4	0.770
HGF	483.4 ± 111.9	313.0 ± 45.8	0.133
IFN-γ	23.2 ± 4.2	16.0 ± 1.8	0.103
IL-10	32.1 ± 10.6	16.2 ± 1.7	0.012
IL-12 (p70)	23.0 ± 4.3	14.6 ± 1.6	0.039
IL-2	50.8 ± 12.0	34.1 ± 3.7	0.093
IL-4	18.9 ± 3.5	13.2 ± 1.5	0.132
IL-5	20.3 ± 4.8	14.4 ± 2.1	0.246
IL-6	25.2 ± 7.5	18.2 ± 2.4	0.270
IL-8/CXCL8	80.1 ± 36.3	37.1 ± 5.5	0.039
CXCL10	1078.6 ± 339.9	631.9 ± 116.2	0.136
CXCL9	345.3 ± 65.8	241.8 ± 32.1	0.178
PDGF-BB	620.6 ± 250.4	171.0 ± 31.6	0.001
SCF	78.9 ± 12.8	48.0 ± 5.3	0.021
SDF-1	876.0 ± 148.1	549.1 ± 54.1	0.020
TGF-β	32.4 ± 6.4	23.2 ± 2.5	0.143
TGF-α	31.6 ± 11.5	16.2 ± 1.8	0.020
TNF-α	22.9 ± 8.0	13.0 ± 1.4	0.042
VEGF-A	94.6 ± 10.2	86.7 ± 17.9	0.841

*SE* standard error

^a^Unpaired t-tests

### Overall survival and serum cytokines

**Fig. 2:**
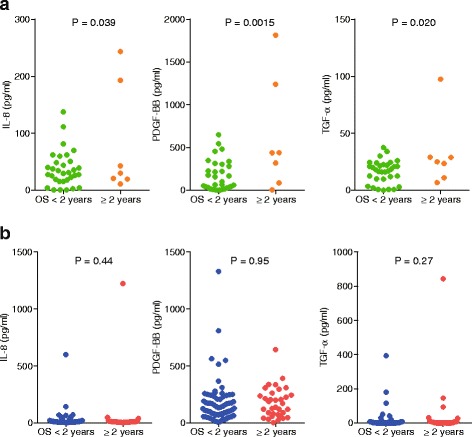
Comparison of serum cytokine levels between long survivors and non-long survivors in the 2 cohorts. **a** In cohort 2 (sorafenib-treated patients), serum IL-8, PDGF-BB, and TGF-α levels were higher in long survivors (overall survival ≥2 years). **b** In cohort 1 (hepatic arterial infusion chemotherapy-treated patients), there were no differences in these serum cytokine levels between long and non-long survivors

Because 3 of the 9 cytokines described above (IL-8, PDGF-BB, and TGF-α) could be used for comparison analysis between cohorts 1 and 2, we further evaluated their expression levels in terms of prediction of overall survival. The remaining 3 cytokines that could be used for comparison analysis (IL-5, CXCL9, and VEGF-A) showed no difference between long survivors and non-long survivors in both cohorts (Additional file 2: Figure S2). Interestingly, we found that serum IL-8, PDGF-BB, and TGF-α were significantly higher in long survivors in cohort 2 (*P* < 0.05) (Fig. 2a) but not in cohort 1 (Fig. 2b).

**Fig. 3:**
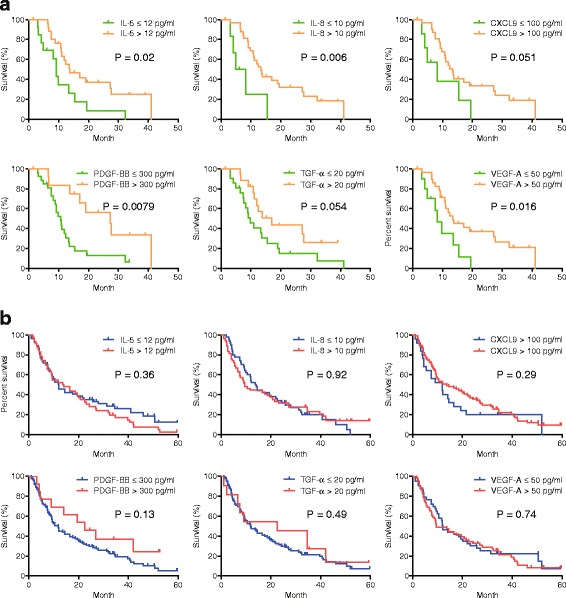
Kaplan-Meier survival analyses of patients with high and low serum cytokines in the 2 cohorts. **a** Sorafenib-treated patients with high serum cytokine levels showed better survival with statistical (IL-5, IL-8, PDGF-BB, VEGF-A) or borderline significance (CXCL9 and TGF-α). **b** Patients who received hepatic arterial infusion chemotherapy showed similar survival rates regardless of serum cytokine levels

We set the cutoff values of 6 serum cytokines that can be used for comparison analysis between cohorts 1 and 2 based on their median values in 143 HCC cases as follows: IL-5 (12 pg/mL), IL-8 (10 pg/mL), CXCL9 (100 pg/mL), PDGF-BB (300 pg/mL), VEGF-A (50 ng/mL), and TGF-α (20 ng/mL). Kaplan-Meier survival analysis showed that patients with high serum IL-5, IL-8, PDGF-BB, and VEGF-A showed significantly better survival when treated with sorafenib; moreover, high CXCL9 and TGF-α levels were also associated with better survival with borderline significance (Fig. 3a). In contrast, serum cytokine levels did not influence survival in patients who received HAIC when using the same cutoff values (Fig. 3b).

**Fig. 4:**
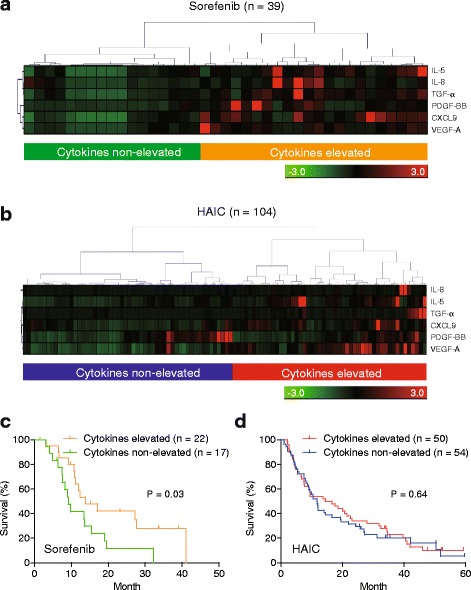
Cytokines profiles and hepatocellular carcinoma (HCC) prognosis. **a** & **b** Hierarchical clustering analysis of 6 serum cytokines profiles. Hierarchical clustering classified HCCs into 2 groups (“cytokines-elevated” and “cytokines-non-elevated”) in a comprehensive and non-arbitrary manner in cohorts 2 (**a**; sorafenib-treated patients) and 1 (**b**; hepatic arterial infusion chemotherapy [HAIC]). **c** & **d** Cytokines-elevated patients showed better survival compared to cytokines-non-elevated patients in cohort 2 (**c**; sorafenib-treated patients) but not in cohort 1 (**d**; HAIC-treated patients)

Next, we incorporated all 6 serum cytokines into a hierarchical clustering analysis to classify HCCs more extensively. Patients were classified into two groups based on the clustering profiles of the serum cytokine, “cytokines-elevated” and “cytokines-non-elevated”, in both cohorts 1 and 2 (Fig. 4a and b). Cytokines-elevated patients showed better survival compared to cytokines-non-elevated patients in cohort 2 (Fig. 4c). In contrast, cytokines-elevated patients showed similar survival rates compared to cytokines-non-elevated patients in cohort 1 (Fig. 4d).

### Potential survival benefit of “cytokines-non-elevated” HCC patients when treated with HAIC

**Fig. 5:**
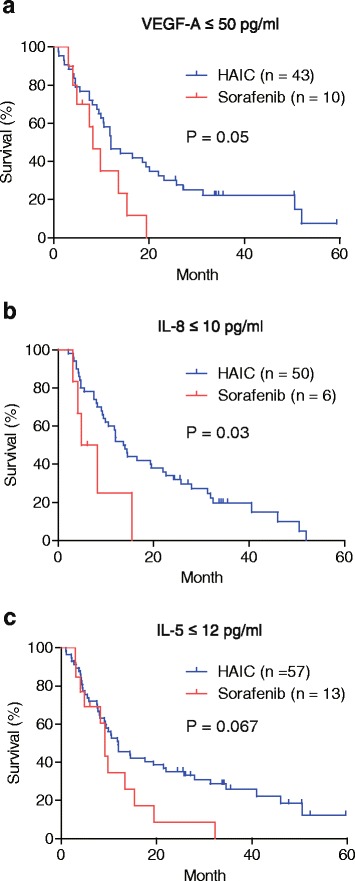
Comparisons of the survival of hepatocellular carcinoma (HCC) patients with low cytokine levels. **a** Kaplan-Meier survival of HCC patients with low serum VEGF-A levels treated with sorafenib or hepatic arterial infusion chemotherapy (HAIC). **b** Kaplan-Meier survival curves of HCC patients with low serum IL-8 levels treated with sorafenib or HAIC. **c** Kaplan-Meier survival curves of HCC patients with low serum IL-5 levels treated with sorafenib or HAIC

Our data suggested that sorafenib is effective in prolonging the overall survival in patients with advanced HCC, with activated stromal cell signaling potentially affecting the serum cytokine profiles. However, sorafenib appears to have limited effects on HCCs without such activation in the tumor microenvironment. We therefore compared the survival rates as related to the 6 serum cytokines in patients who received sorafenib and HAIC to evaluate the effects of HAIC on HCC absent the activated stromal cell signaling. In cases of HCCs with low serum VEGF-A (Fig. 5a) (≤50 pg/mL, represented by 43 of 104 patients treated with HAIC and 10 of 39 treated with sorafenib), IL-8 (Fig. 5b) (≤10 pg/mL, represented by 50 of 104 patients treated with HAIC and 6 of 39 treated with sorafenib), and IL-5 (Fig. 5c) (≤12 pg/mL, represented by 57 of 104 patients treated with HAIC and 13 of 39 treated with sorafenib), patients with low VEGF-A and IL-8 who received HAIC exhibited significantly better survival compared to those treated with sorafenib; those with low IL-5 also showed better survival with borderline significance. In cases of low serum TGF-α (≤20 pg/mL), PDGF-BB (≤300 pg/mL), and CXCL9 (≤100 pg/mL), HCC patients who received HAIC showed similar survival rates compared to those treated with sorafenib (Additional file 3: Figure S3).

## Discussion

Taken together, our findings suggested that the selected serum cytokines identified herein may be useful biomarkers for predicting HCC patients who may achieve survival benefits owing to sorafenib treatment. Sorafenib is widely used for the first-line treatment of advanced HCC patients worldwide. However, it has been difficult to identify HCC patients who will achieve survival benefits owing to sorafenib treatment. Several previous studies demonstrated that genomic alterations such as *VEGFA* or *FGF3/4* amplification can identify HCC patients who showed clinical responses after sorafenib treatment, although tumor biopsy specimens are required. Herein, we showed for the first time that evaluation of selected serum cytokines may be useful for identifying HCC patients with potentially active microenvironmental signaling that may increase sensitivity to sorafenib. Interestingly, the same cytokines were not useful for identifying HCC patients who may benefit from HAIC, potentially because of the different cell populations targeted by HAIC (which mainly S-phase tumor epithelial cells) and sorafenib (which mainly targets the stromal vascular endothelial cells).

We identified three cytokines (HGF, IL-4, and SDF-1) that are differentially elevated in clinical responders compared with non-responders; among them, only serum IL-4, a Th2 cytokine, could discriminate HCCs according to overall survival. Because Th2 cytokines may contribute to worse prognosis in HCC patients who underwent surgery, it is possible that Th2 cytokines may somehow influence the tumor responses to cytotoxic reagents [12]. Further studies are required to evaluate the role of Th2 cytokines and their effects on HCC phenotypes in terms of chemoresistance.

We found that serum IL-5, IL-8, CXCL9, PDGF-BB, TGF-α, and VEGF-A were elevated in the long survivors group among HCC patients who received sorafenib compared to those who received HAIC. Because sorafenib is known to target multiple receptor tyrosine kinases such as PDGF receptors, VEGFRs, and EGF receptors, it makes sense that elevation of the serum cytokines PDGF-BB, TGF-α, and VEGF-A could reflect the activation status of microenvironmental signaling pathways that may correlate with survival benefits achieved by sorafenib [13, 14]. However, it was unclear if sorafenib directly targets the IL-8 receptor CXCR1/2, IL-5 receptor, and CXCL9 receptor CXCR3. It is possible that these cytokines may indirectly activate the known receptor tyrosine kinases targeted by sorafenib; future studies are required to test these hypotheses.

We detected differences in vascular invasion and metastasis profiles between cohorts 1 and 2. Although the reasons for these differences were unclear, they may be attributed to the fact that sorafenib is frequently used to treat patients with advanced HCC who have distant organ metastasis, while HAIC mainly targets intrahepatic lesions and is considered ineffective against metastatic lesions.

This study has some limitations. First, it was performed at a single institution. Second, the profiles of the patients in the 2 cohorts were different in terms of the frequency of vascular invasion and distant metastasis. Third, the sample size of the patients who received sorafenib was small; hence, the statistical outcomes of this analysis should be validated in an independent cohort, which we are currently recruiting the patients. Fourth, our two HCC cohorts may have been subject to selection bias, which might make the treatment outcomes as determined by cytokine evaluation uncertain. Fifth, samples were only evaluated at a single time point (baseline); serial measurements may be more useful for predicting potential benefits. Future prospective studies are required to address these limitations by recruiting more patients in a multicenter setting using the same protocols.

## Conclusion

Patients who exhibited survival benefits by sorafenib or HAIC showed different pre-treatment cytokine profiles. Evaluation of the serum cytokines investigated in our study may be useful to predict responses in advanced HCC patients who receive sorafenib, which mainly targets stromal cells such as vascular endothelial cells. Our data also suggested that cytokines have limited roles in predicting HCC responses in patients who receive HAIC, which mainly targets cancer epithelial cells. Hence, our findings expose important biomarkers in advanced HCC patients that can predict survival benefits owing to sorafenib or HAIC prior to the treatment.

## Additional files

Additional file 1: Figure S1. Kaplan-Meier survival analysis of HCC patients classified as HGF-high and -low, IL-4-high and -low, and SDF1-high and –low in Cohort 2. (PDF 101 kb)

Additional file 2: Figure S2. Comparison of serum IL-5, CXCL9, and VGEF-A levels between long survivors and non-long survivors in the 2 cohorts. (PDF 117 kb)

Additional file 3: Figure S3. Kaplan-Meier survival analysis of HCC patients with low serum TGF-α (≤20 pg/mL), PDGF-BB (≤300 pg/mL), and CXCL9 (≤100 pg/mL). (PDF 117 kb)

## Abbreviations

AFP: Alpha-fetoprotein
AFP-L3: Lectin-reactive AFP
CXCL: Chemokine (C-X-C motif) ligand
CXCR: C-X-C chemokine receptor
DCP: Des-gamma carboxyprothrombin
EGF: Epidermal growth factor
ELISA: Enzyme-linked immunosorbent assay
FGF: Fibroblast growth factor
HAIC: Hepatic arterial infusion chemotherapy
HCC: Hepatocellular carcinoma
HGF: Hepatocyte growth factor
IFN: Interferon
IL: Interleukin
PDGF: Platelet-derived growth factor
SCF: Stem cell factor
SDF1: Stromal cell-derived factor 1
TGF: Transforming growth factor
TNF: Tumor necrosis factor
VEGF: Vascular endothelial growth factor
VEGFR: Vascular endothelial growth factor receptor
