# Supplementary figures and images for: Serum cytokine profiles predict survival benefits in patients with advanced hepatocellular carcinoma treated with sorafenib: a retrospective cohort study
Source: BMC Cancer. 2017 Dec 19;17:870. doi: 10.1186/s12885-017-3889-x (PMC5738185; doi:10.1186/s12885-017-3889-x)

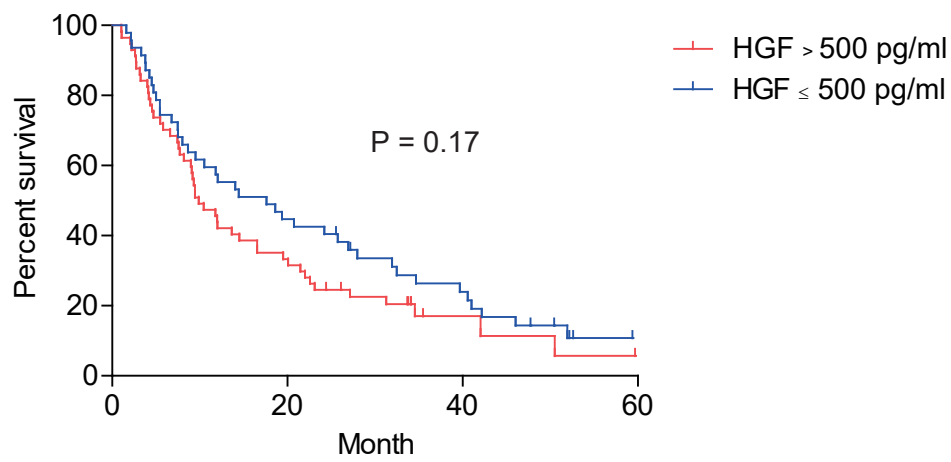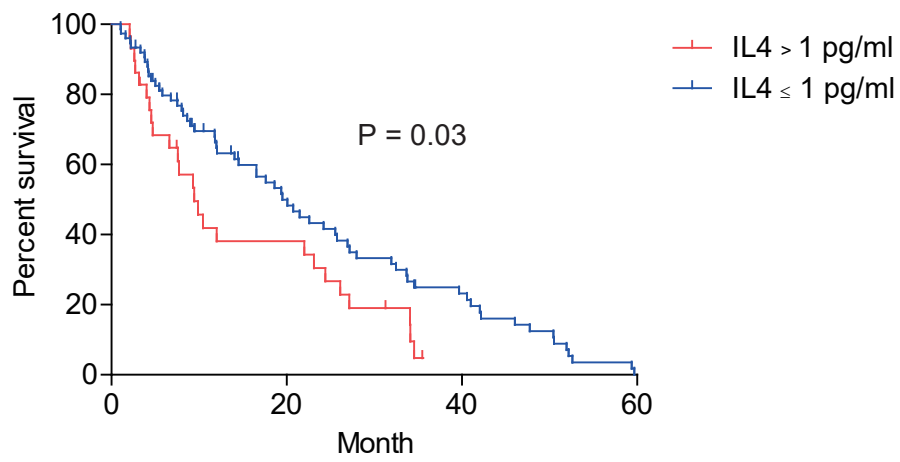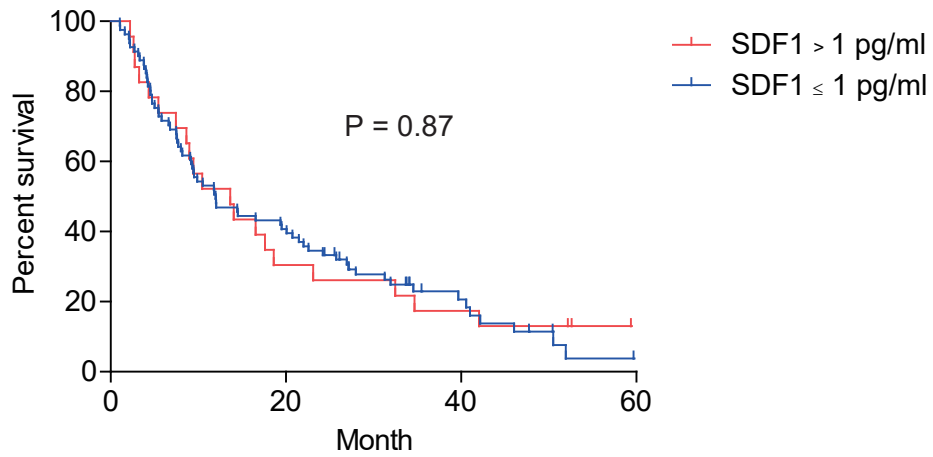

Supplement: Supplementary file 1 — Kaplan-Meier survival analysis of HCC patients classified as HGF-high and -low, IL-4-high and -low, and SDF1-high and –low in Cohort 2. (PDF 101 kb) [file 12885_2017_3889_MOESM1_ESM.pdf]

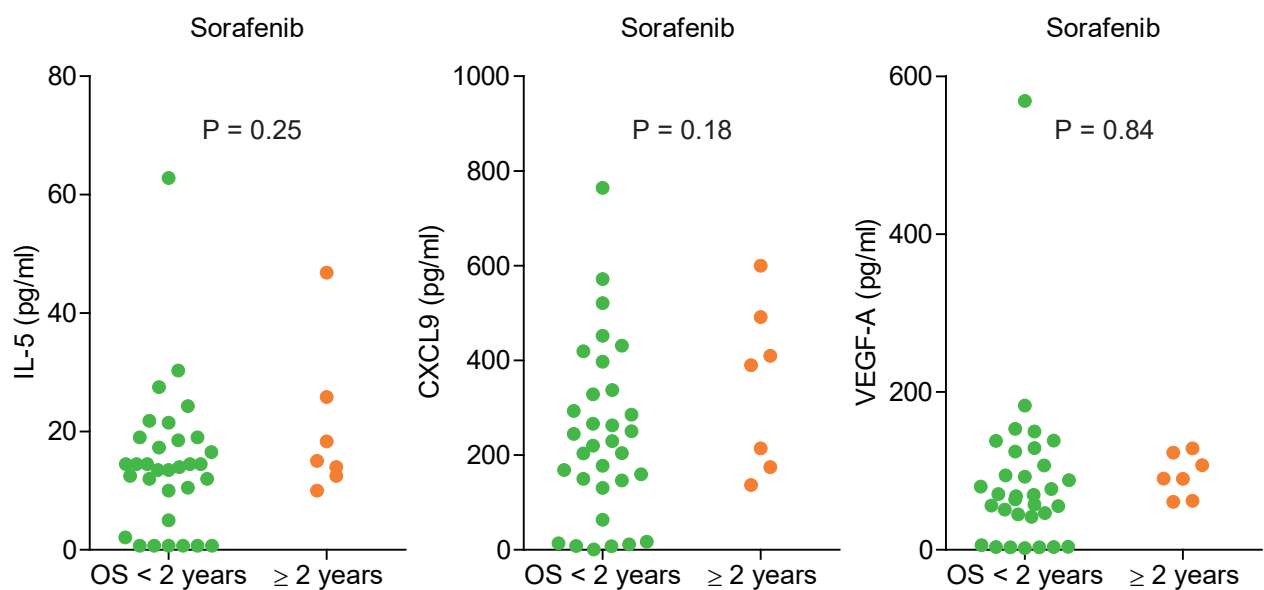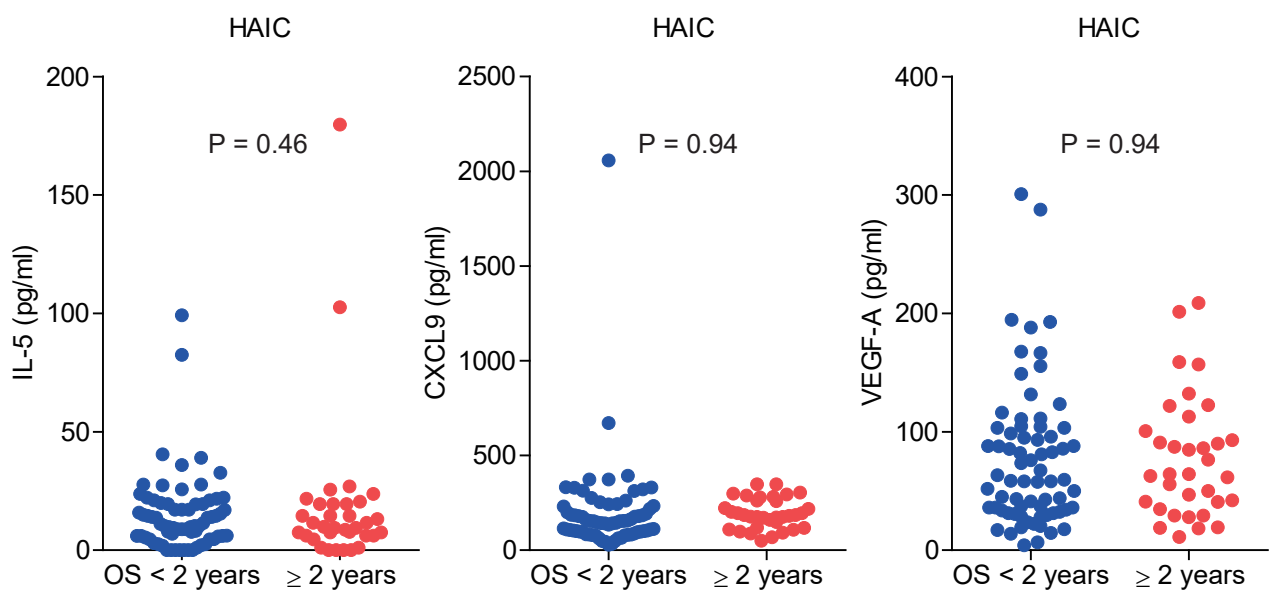

Supplement: Supplementary file 2 — Comparison of serum IL-5, CXCL9, and VGEF-A levels between long survivors and non-long survivors in the 2 cohorts. (PDF 117 kb) [file 12885_2017_3889_MOESM2_ESM.pdf]

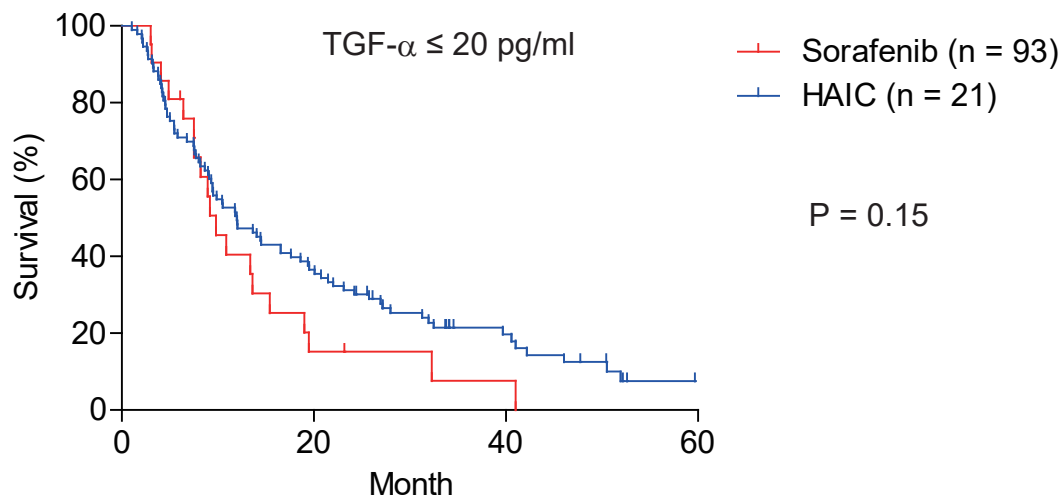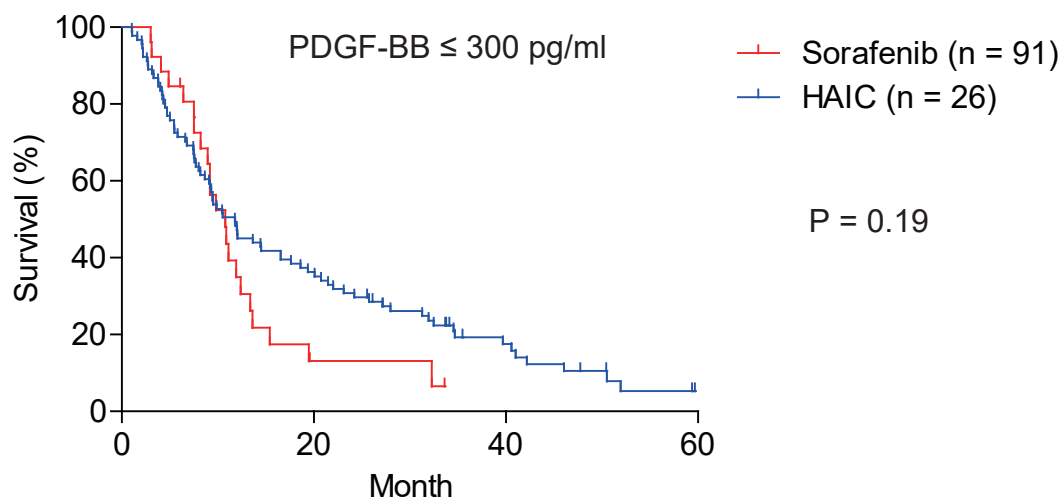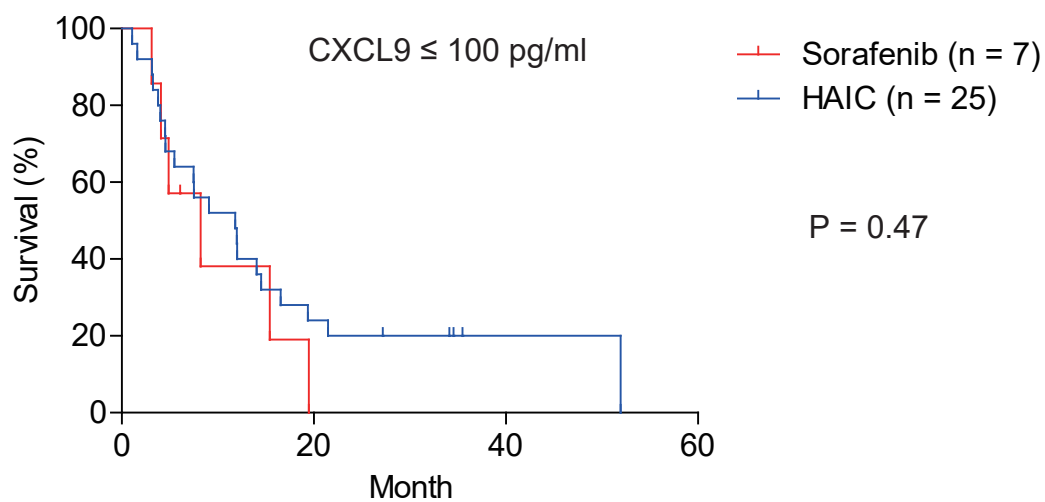

Supplement: Supplementary file 3 — Kaplan-Meier survival analysis of HCC patients with low serum TGF-α (≤20 pg/mL), PDGF-BB (≤300 pg/mL), and CXCL9 (≤100 pg/mL). (PDF 117 kb) [file 12885_2017_3889_MOESM3_ESM.pdf]
